# CEST MRI in the Management/Diagnosis of Neuroinfectious Diseases

**DOI:** 10.3390/ijms26125650

**Published:** 2025-06-12

**Authors:** Zoe A. Kortje, Horacio Bach

**Affiliations:** 1 Faculty of Medicine, University of British Columbia, Vancouver, BC V6T 1Z3, Canada; 2 Department of Medicine, Division of Infectious Diseases, University of British Columbia, Vancouver, BC V6H 3Z6, Canada

**Keywords:** CEST, MRI, neuroimaging, infectious diseases

## Abstract

Chemical exchange saturation transfer (CEST) MRI is a novel technique that allows for the specific imaging of certain molecules that contain exchangeable protons. Neuroimaging is a major contributor to diagnosing and monitoring infections of the central nervous system (CNS). This review focuses on summarizing the current literature surrounding the use of CEST MRI imaging in diagnosing, monitoring, and treating CNS infections. BacCEST is a new technique to detect bacterial infection in organs at profound levels. This technique allows for the specific pathogen causing the infection to be understood, allowing for tailored antibiotic therapies. The bacCEST signal is also directly proportional to the number of bacterial cells; this means it can be used over periods to track response to treatment via cell numbers. The results show that most of the research in this area has focused on infections of the brain parenchyma (e.g., encephalitis) and that most studies investigate the use of CEST in animal models, with a minority exploring the application of CEST to human participants. The common neuroinfectious disease presentations relevant to clinical medicine are also briefly described, as well as the traditional and modern imaging techniques used to visualize them.

## 1. Introduction

**Figure 1:**
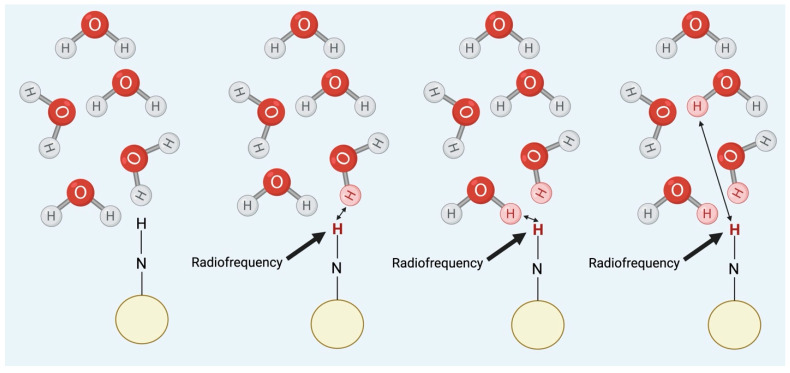
CEST mechanism. Radiofrequency is used to saturate an amine group present in a substance, which reduces the signal of that substance. As a result, a saturated hydrogen proton is transferred to water. The process then continues with a concurrent reduction in the water signal. Adapted from [1]. reated in BioRender. Bach, H. (2025) https://BioRender.com/h42n006.

Chemical exchange saturation transfer (CEST) MRI is a novel technique that allows for specific imaging of certain molecules containing exchangeable protons. These agents can be either exogenous (administered before imaging through injection) or endogenous, as the body’s naturally occurring compounds (e.g., amines, amides, and hydroxyl groups) also contain exchangeable protons when they become saturated at specific resonance frequencies (Figure 1) [1]. CEST MRI was first characterized by Wolff and Balaban in 2000, resulting from a combination of established processes of magnetization transfer and chemical exchange [1]. This imaging technique has been explored through numerous preclinical and clinical research studies within various pathophysiological processes. These studies have demonstrated that, in many cases, CEST MRI exhibits increased sensitivity compared to magnetic resonance spectroscopy (MRS) and conventional MR imaging due to its ability to image certain compounds, even at very low concentrations [1].

## 2. CNS Infections

CNS infections continue to pose a significant burden on healthcare systems worldwide. A recent multinational study found that 27.3% of patients with CNS infections worldwide experienced unfavorable outcomes, including persistent disability and death [2]. Neuroimaging is a major contributor to the diagnosis and monitoring of infections of the CNS. Structural (e.g., MRI/CT) and molecular techniques (e.g., PET) have been utilized for diagnosis and monitoring therapy response in these cases [3]. Despite the utility of these current techniques, structural imaging tends to be nonspecific and, in many cases, cannot differentiate infectious lesions from other pathologies, such as cancerous or ischemic lesions [3]. Molecular imaging through PET requires the usage of exogenous radiotracers, leading to challenges due to expense, clinical availability, radiation exposure, and the need for these molecules to cross the blood–brain barrier [1]. Conversely, CEST contrast can be generated through endogenous means without ionizing radiation and with high spatial resolution [4]. These advantages suggest that CEST could be a promising new MRI technique for visualizing CNS infections.

This review will summarize the existing literature on the use of CEST MRI imaging for diagnosing, monitoring, and treating CNS infections. It will outline common CNS infections and their current diagnostic tools, followed by a brief overview of CEST MRI physics and the various CEST agents being explored for clinical use.

The cause, presentation, and pathophysiology of infections affecting the CNS (e.g., the brain and spinal cord) vary considerably. The three main infective processes of the CNS include encephalitis (infection of the brain’s parenchyma), meningitis (an infection of the meninges), and brain abscess (a focal infection that forms a capsule around a collection of pus) [5].

Microorganisms implicated in meningitis and encephalitis cause infection by direct brain invasion following initial mucosal colonization. Meningitis can also occur through the hematogenous spread of pathogens or direct inoculation via neurosurgical procedures [5]. Brain abscesses form through the direct entry of pathogens into the brain during procedures or from previous infections in the teeth, sinuses, or ears. Abscesses can also be seeded through hematogenous spread from infection in other organs, while many have no identifiable cause [5]. Other less common infections of the CNS include epidural abscess, subdural empyema (most commonly located over the cerebral convexity), and ventriculitis. Bacteria, viruses, fungi, and parasites can propagate these infections [6]. The specific microbiological causes of each of these processes are beyond the scope of this project; for this information, we refer readers to the reviews by Refs. [7,8].

## 3. Imaging of CNS Infections

Diagnosing CNS infections requires the compilation of multiple streams of evidence, including clinical evaluation, cerebrospinal fluid (CSF) samples, tissue biopsy, and neuroimaging [6]. Briefly, a CSF sample is obtained through a lumbar puncture or shunt tap, which is contraindicated in patients who have a higher risk of increased intracranial pressure or a mass lesion, as this may cause uncal or transtentorial herniation [9]. The CSF parameters most effective in differentiating the pathogenic cause of a CNS infection are glucose, protein, and cell count (with differential). A CSF Gram stain is also helpful for diagnosing bacterial infections as it detects positive cultures in 80% and organisms in 60–90% of cases [10]. Neuroimaging of CNS infections allows for the recognition of classic lesion patterns, contributing to patient diagnosis and the ability to monitor treatment response and patient recovery [11].

Given that this review focuses on a novel technique for the neuroimaging of CNS infections, it will also discuss in detail the primary modalities of the image-based investigation currently used in clinical practice.

### 3.1. CT

CT is the first-line imaging modality in acute and uncomplicated CNS infection. It is often completed before obtaining a CSF sample to rule out the possibility of a mass lesion [6]. This is because these scans can exclude critical findings such as cerebral edema, hydrocephalus, and other skull findings (e.g., inner ear pathology and craniofacial and temporal fractures) [6,12]. These skull data from initial CT images can be essential in meningitis cases to determine the entry point or source of the CNS infection [11]. The injection of CT contrast can allow for better visualization of inflammation, edema, abscess, and ischemia [12]. CT venography is another adjunct modality that visualizes cerebral venous sinus thrombosis (a potential complication in meningitis), particularly in the sagittal and transverse sinuses [11]. CT imaging is, therefore, a solid option for the efficient (and affordable) imaging of initial CNS infection and is most commonly useful in cases of bacterial meningitis [11]. However, this technique may not identify other types of CNS infection (e.g., abscess, subdural empyema, and viral meningoencephalitis) and earlier pathological changes due to its inferior soft tissue resolution [13].

### 3.2. MRI

Conversely, MRI is the most sensitive technique for diagnosing CNS infection [14]. Primary MR sequences can delineate detailed neurophysiological tissue abnormalities (including inflammation and ischemia), while many other functional MR techniques have been invented to expand on this capability. Most infections in the brain can be characterized by T1, T2, and gadolinium (Gd)-enhanced imaging sequences, which allow for pattern recognition and broad categorization of the infection [14]. Other sequences commonly visualized through MRI include fluid-attenuated inversion recovery (FLAIR), which decreases the CSF signal for better visualization of “white” pathology, and diffusion-weighted imaging (DWI), which creates contrast through measuring water diffusion in parenchyma [15]. The latter is often used to differentiate brain abscesses from tumors since water molecules have limited free motion in the necrotic center of an abscess [15]. Lastly, diffusion kurtosis imaging (DKI) is another complementary MRI measure that extends from DWI, which provides kurtosis tensor (DK)-derived metrics; these can quantify how water diffusion differs from the Gaussian distribution and, therefore, can estimate the degree of diffusion restriction [16].

Magnetic resonance spectroscopy (MRS) provides functional, instead of anatomic, images through the same physics principles as MRI. It is a non-invasive technique that can measure the concentrations of chemical compounds within tissues. These pieces of data are presented on an MR spectrum, which contains peaks corresponding to the metabolites detected within the tissue [17]. Specific compounds at certain resonance frequencies can provide information about infections and lesions in the CNS; for example, the ratio of acetate/succinate (each resonating at a different frequency) can delineate parasitic from pyogenic infections [17].

### 3.3. Molecular Techniques

Many other techniques have been created and investigated in animals for imaging CNS infection. A few of them will be briefly characterized here. Nuclear imaging technologies, including PET (positron emission tomography) and SPECT (single photon emission computed tomography), have very high sensitivity and spatial resolution when visualizing neuroinflammation [18]. These techniques utilize signal molecules (radioisotopes) injected into the subject. They can present neuroinflammatory processes in several ways, including changes in vascular permeability, glucose metabolism, and leukocyte infiltration [18]. Optical imaging techniques (such as bioluminescence and near-infrared fluorescence imaging) have also been successfully applied to study CNS infection. For example, a luciferase-transfected pathogen was used to study the real-time development of African trypanosomiasis infection in mice using in vivo bioluminescence imaging [19].

### 3.4. Chemical Exchange Saturation Transfer (CEST)

Chemical exchange saturation transfer (CEST) is another type of molecular imaging technique created for use in MRI. The physics of standard MR imaging utilizes the excitation of hydrogen molecules to generate high-contrast images of bodily structures’ anatomy and pathological processes. Conversely, CEST is a sensitivity enhancement mechanism that uses the excitation of other solutes (i.e., metabolites and chemical compounds) to visualize these images [18,20]. In this technique, a biomolecular group possessing a chemical shift (a radiofrequency difference in radians per second) distinct from water is selectively saturated, causing the associated protons to display no net magnetization [21]. Then, this magnetization is shifted from these target protons to the bulk water protons via chemical exchange, causing the water signal to decrease in the associated MRI images and the target species to become visible [1]. CEST is a continuous cyclical process with consistent saturation of metabolite protons (through the application of the correct resonance frequency) and, therefore, constant transfer of this magnetization from the target metabolite to water until a steady state chemical reaction is reached [1]. This allows for the species of interest to be visualized even when it exists at very low concentrations; this can provide information about various pathophysiological processes. In addition to the concentration of the compound, the chemical exchange depends on other collateral factors such as the proton exchange rate, surrounding pH, and temperature [1]. For CEST to be optimized, the proton exchange rate of the target compound should be between slow and intermediate on the NMR time scale; the exchange rate must be less than the substance’s chemical shift difference from water for these two species to be differentiated in the MRI image [22]. Overall, the bulk water signal reduction in CEST imaging occurs as a function of the concentration of exchangeable protons associated with each CEST agent, the endogenous T1 relaxation time of the tissue water protons, the RF saturation power (amplitude of the RF pulse), its duration (how long the RF pulse is applied), and the proton exchange rate [22]. The enhancement of the solute depends on the proton concentration and the exchange rate [1].

## 4. Types of CEST

There are many variations of chemical exchange saturation transfer based on the isolated chemical compound. The main requirement for this technique is that the target substance possesses an exchangeable ^1^H proton and that the solute in which CEST occurs has abundant water for this exchange to occur [1]. There are two main classification paradigms for the different variations of CEST imaging; one relies on chemical shift patterns, while the second classifies by what type of exchange is occurring (i.e., proton, molecular, or compartmental) [1]. This second organizational system is still in the early stages; other approaches include CEST techniques for the specific mechanism involved (i.e., glycoCEST for glycogen transfer) [20]. Given that the chemical shift system is the most well-established and clinically applicable, these classifications will be used in this review and subsequently explained.

This denomination divides CEST molecular imaging using the terminology of diaCEST and paraCEST, which encompass the use of this technique with diamagnetic and paramagnetic species, respectively [23]. The agents in both groups utilize a similar mechanism by creating negative contrast in MR images as the protons bound to the CEST compound are shifted away from the frequency of the body’s water [22]. The division between these two groups depends on their difference in chemical shifts compared to water. Paramagnetic compounds are typically exogenous, contain metallic ions, and have a much more significant frequency shift than their diamagnetic counterparts. This allows the proton exchange rate to be much faster in these compounds, even with standard slow–intermediate MR exchange and therefore supports the imaging of faster-exchanging species [20]. Diamagnetic compounds do not contain metallic ions, can be endogenous, and have a closer chemical shift to water (typically 0–7 ppm from water) [1,20]. Some research teams have attempted to classify diaCEST and paraCEST agents into subgroups; the main findings from this endeavor will be discussed below.

### 4.1. Paramagnetic Agents

Paramagnetic agents can be subdivided based on the location of the mobile protons within the system. True paraCEST agents contain the mobile protons critical to the physics of CEST imaging. Their chemical makeup consists of lanthanide (III)-based metal complexes, which may be associated with water protons chelated to the Ln (III) or with mobile protons belonging to a ligand [24]. The primary ligands used for paraCEST agents are macrocyclic tetra-amide derivatives of DOTA (dodecane tetra-acetic acid), a complexing agent featuring four carboxylic groups commonly employed in theranostics due to its strong chelation properties and thermodynamic stability [25]. DOTA’s four carboxylate groups are crucial in providing the Ln (III) complex with a single negative charge, which enhances its tolerability in vivo [24]. These chemical groups contribute the most electron density to the lanthanide ion, increasing the rate of water exchange. This can create challenges in distinguishing the signal of the paramagnetic agent from the background signal emitted by the water molecules [22]. Therefore, ligands containing amide groups (e.g., DOTA-tetraamides) are more effective paraCEST agents since the oxygens in amide groups are poorer electron donors, and the proton exchange rate is slower (26).

Another genre of paramagnetic agents is called supraCEST, which describes paramagnetic agents that have been adducted onto a substrate that acts as a source of mobile protons [24]. This technique improves the sensitivity of these agents, likely through an increase in shift difference and decreased exchange rate speed through this interaction [24].

### 4.2. Diamagnetic Agents

The performance of diamagnetic substrates differs based on the molecular weight of the targeted compound, prompting the designation of low-molecular-weight (LMW) diaCEST and macromolecular diaCEST. The first CEST imaging used NH_4_Cl, which became the first of the LMW CEST agents [24]. Indole and pyrimidine rings, featuring their heterocyclic NH group, are promising LMW CEST compounds, while iopamidol is a more recently discovered CEST agent with improved sensitivity [26]. Macromolecular diaCEST was developed due to the enhanced CEST sensitivity in the presence of more mobile protons from cationic polymers [27]. The most effective of these, concerning the CEST effect, is poly-lysine and the G-5 PAMAM dendrimer. Notably, the adduction of the dendrimer with polyuridilic acid has facilitated CEST MRI visualization of gene therapy (24).

Lastly, using endogenous molecules as diaCEST agents was first demonstrated in vivo through imaging of the human kidney [28]. By optimizing the visualization of these metabolites under physiological conditions, it is possible to conduct non-invasive and non-ionizing investigations across multiple organ systems [29]. Endogenous CEST agents that have been studied include proteins, glucosaminoglycans, glycogen, myoinositol, glutamate, and creatinine [29]; these can help detect their presence as well as that of other compounds, while also enabling the imaging of environmental factors such as pH, temperature, and transplanted cells [30]. The basic parameters and clinical applications of well-established endoCEST strategies will be discussed below.

### 4.3. EndoCEST

Amide proton transfer (APT) is one of the most well-known CEST imaging techniques. Amide protons have a slow chemical exchange rate, allowing clear contrast in CEST images. Amide protons are also found in all tissue types because of their integral presence in proteins [31]. The slow rate of proton exchange in APT allows this technique to be used at 3 T and higher magnetic fields, and developments within the field show significant promise for its translation to clinical practice [29]. APT has explored how CEST with amide protons can detect pH decreases, manifesting as a reduction in CEST contrast on MR images and indicating early brain ischemia before T1, T2, or diffusion-weighted images can discern this condition [20]. APT has also been successfully applied to human subjects, where APT imaging has differentiated between brain tumors, edema, and normal white matter [32]. Additional applications of APT include visualizing malignant cells based on increased concentrations of cellular protein/peptide [23] and using pattern recognition to detect non-enhancing high-grade tumors [32].

CEST involving –OH exchange has been investigated with endogenous molecules such as glycogen, glucose, and glucosaminoglycans. The contrast generated by –OH exchange is less sensitive than other CEST methods because of the lower frequency separation of –OH from water, which does not fall within the slow–intermediate exchange on the NMR time scale [29]. The promise of higher field scanning (i.e., 7 T) will make this form of CEST more practically applicable, allowing more separation between the chemical shift of hydroxyl and bulk water protons. Nevertheless, glycogen CEST (glycoCEST) has been proven effective in animal studies with 4.7 T scanners [23]. The importance of glycogen in the liver has prompted the idea that this type of imaging may be helpful in the diagnosis of diseases impacting glycogen metabolism, such as diabetes and obesity [29]. Glycosaminoglycans (GAGs) have also been considered a CEST agent because of their roles in many crucial physiological functions. For example, GAGs (and their associated proteoglycans) are primarily implicated in cartilage health and associated diseases such as osteoarthritis [33]. Every GAG compound has one amine and three hydroxyl groups; the exchange of protons of the –OH side chains allows for quantifying GAG concentration changes in vulnerable cartilaginous areas through CEST imaging techniques [33]. Lastly, glucose CEST (glucoCEST) was first pioneered in 2012 and has mainly been examined in neuro-oncology. D-glucose injection into the brain allows for CEST visualization of tumors due to the difference in pH between the tumor and surrounding parenchyma (and the pH-dependent effect of CEST) [34]. This mechanism also allows glucoCEST to delineate between two cancer cell lines within the mass, which other MRI imaging techniques cannot characterize [35]. Other studies have demonstrated that phosphorylated glucose levels can detect cerebral metabolism [36] and neuronal activation (via correlation of glucoCEST with BOLD fMRI) [37]. This imaging technique, in combination with APT and DWI, has demonstrated high sensitivity (95.4%) and specificity (87.18%) for the diagnosis of rectal cancer demonstrating its potential as a diagnostic imaging marker.

The exchange of amine protons is also possible for CEST application. The most common endogenous agents with this chemical side chain are glutamate and creatinine, which have been investigated in various pathologies [29]. Owing to the critical role of glutamate in neural metabolism and communication, glutamate imaging (gluCEST) has been mainly studied in diseases involving the central and peripheral nervous systems. For example, it has been found that gluCEST imaging can provide detailed glutamate metabolism mapping throughout different areas of the brain [38] and can contribute to imaging neuroinflammatory responses such as those involved in traumatic brain injury and cerebral artery occlusion in humans [39]. Creatinine (CrCEST) is often used to monitor muscle energetics because it is a by-product of ATP production during exercise [29]. The exchange rate of creatinine (around 1.8 ppm from water) is directly proportional to the creatinine concentration at bodily pH. It can be separated from other metabolites, allowing CrCEST to provide information on muscle pH, creatine kinase kinetics, and oxidative metabolism. This has shown clinical utility in investigating and understanding cardiac and skeletal muscle disorders [29].

BacCEST has been a significant breakthrough for CEST in studying infectious diseases. The current MR methods used in the clinic (i.e., contrast-enhanced, DWI, dynamic susceptibility contrast perfusion-weighted imaging) are not specific to CNS infection. They cannot differentiate brain abscesses/encephalitis by etiology [40]. BacCEST uses the natural CEST contrast created by bacteria to detect infection in areas of the body that contrast agents may not reach (e.g., CNS, bone marrow). This technique allows for the specific pathogen causing the infection to be understood, allowing for tailored antibiotic therapies. The bacCEST signal is also directly proportional to the number of bacterial cells; this means it can be used over periods to track the response to treatment via cell numbers [40].

CryptoCEST is another CEST technique that utilizes the metabolite trehalose disaccharide produced in high amounts by cryptococcal cells [41]. This compound corresponds to a resonance frequency of 0.2–2 ppm, created by the proton within its –OH that can exchange with bulk water in the same way as glucose, glycogen, and other –OH-containing chemical groups. The endogenous trehalose concentration in cryptococcomas exceeds those of these comparable glucose molecules by 2-fold; thus, the exchange inside the fungal lesion creates a CEST contrast that allows for discrimination from normal brain tissue at 0.7 ppm [41]. This technique has allowed the identification of cryptococcomas smaller than 1 mm^3^, which is more sensitive than MRS because of the diluting partial volume effect required in the latter. It has also been successfully utilized to differentiate cryptococcal lesions from gliomas (which are not visible at 0.7 ppm CEST) to a more reliable extent than T2W MRI [41].

### 4.4. CEST Theranostics

CEST techniques have also been created for use in the central nervous system through theranostics. Typically, theranostics requires that an exogenous imaging agent is attached to the drug being administered or that the drug and agent are packaged together in a typical nanoparticle [42]. However, these methods face numerous limitations in their usefulness and safety, mainly when applied to the brain. As mentioned above, the nanoparticles’ loading rate can be too slow to allow for effective therapy concentrations or adequate sensitivity for imaging this process [43]. In addition, the exogenous particles used in these techniques can be toxic to the patient or may not be able to cross the blood–brain barrier, a unique challenge faced by theranostic techniques for the CNS. Diamagnetic CEST contrast agents do not require injection as they emerge from the intrinsic properties of molecules with exchangeable protons [20]. By determining the CEST contrast of specific medications for the CNS, it is possible to simultaneously image their distribution throughout the brain as they work to target the infection at hand [42]. Due to the biophysical parameters of this imaging technique, this application only works with drugs that contain slow to intermediate exchanging protons [44]. For example, the recent creation of CEST contrasts based on the antiretroviral therapies lamivudine and emtracibine can target the neurocognitive sequelae of HIV [42].

## 5. Summary

To summarize, there are many types of CEST that have been created since the technique’s inception in 2000. Categorization of these variations is helpful to clarify their mechanisms and similarities; the main denomination currently used in the field divides CEST agents into paramagnetic and diamagnetic. These classify the agents based on their proton chemical shift from the frequency of water in human body compartments. Diamagnetic compounds include the large subcategory of endoCEST, which uses endogenous molecules within the body as the CEST substrate for imaging. Lastly, CEST concepts have also been applied to theranostics, where the shift of the protons within a therapeutic compound allows it to be tracked. The types of CEST discussed above are summarized below (Table 1).

## 6. Methods

The approach to developing this review was a literature search for preclinical and clinical studies published in peer-reviewed journals since 2000 (when CEST imaging was first characterized). This research primarily aimed to investigate the potential of CEST MRI for clinical application in the diagnosis, monitoring, and treatment of neuroinfectious diseases. For this project, the infections included in this category were meningitis, encephalitis, meningoencephalitis, abscess, subdural/epidural empyema, ventriculitis, and sepsis-associated encephalopathy (SAE). This review involved a search of both Google Scholar and PubMed using predetermined keywords. The following keywords were used to find relevant articles: “CEST” OR “CEST MRI” AND “Infection” AND “CNS” OR “Brain” OR “Spine”. No previous review has been completed on this topic.

The inclusion criteria required that the paper focus on a clinical application of CEST MRI, that the application be directly related to one or more types of neuroinfectious disease, and that the study investigated this technique in animal or human subjects (not just cell lines). Papers were excluded if they were not in English, were case reports, or were duplicates (many papers were found in both databases). Papers discussing the visualization of inflammation in the CNS due to a non-infectious etiology (e.g., autoimmune encephalitis) were also excluded for clarity.

Data extraction was performed by focusing on specific attributes of the selected studies. These were the author(s), year published, subjects, CNS infection, MRI scanner type, CEST type, CEST application, and research outcomes. The findings for each article review have been synthesized into a table in the discussion section.

## 7. Results

This review assessed the current literature on using CEST MRI imaging for diagnosing, monitoring, and treating neuroinfectious disease. The literature search yielded eight preclinical and three clinical studies directly investigating CEST MRI in one of these applications. The neuroinfectious diseases which were examined in these studies included brain abscess, encephalitis, and sepsis-associated encephalopathy. No papers were found that applied CEST MRI imaging to other infections of the CNS; namely, there were no significant findings for meningitis, spinal diseases, or epidural/subdural abscesses. The papers found through this literature review used many variations of CEST MRI imaging, whose mechanisms were described in detail in the introduction. The most common type of CEST used to image neuroinfectious diseases in this review was gluCEST (five publications). Notably, most clinical trials (2/3) used amide CEST (APT imaging). There was also a range of MRI scanners used in these papers, with the lowest strength measuring 3 T (used in clinical studies) and the highest measuring 11.7 T (used to image bacCEST MRI in rats). The studies included in this review were all published within the past ten years.

### 7.1. Preclinical Findings

The papers included in this review used cellular and animal models to investigate how CEST MRI can be used as a tool in the field of neuroinfectious disease (Table 2). Many focused on how CEST techniques can be used to improve the efficiency and accuracy of the diagnosis of these diseases. A study with bacCEST found that this imaging technique provided better contrast in *S. aureus* abscesses (in F344 Fisher rats) than injectable alternatives used in conventional MRI, as the latter only achieved poor perfusion of these lesions [45]. By comparing the CEST contrast generated by *S. aureus* to another bacterial strain (*Clostridium novyi*-NT), the study found that the endogenous bacCEST signals were significantly different, suggesting that future diagnostic CEST methods could differentiate the bacterial cause of brain abscess based on the CEST signal observed [45].

In particular, CEST has significant diagnostic potential in differentiating infective pathogenesis in the CNS from other lesions (e.g., ischemic, neoplastic). The bacCEST study [45] was able to differentiate brain abscesses and tumors, which is currently unreliable with conventional MRI techniques. Still, a statistically significant difference between the abscess and tumor signal was recorded using different B1 values (1 μT and 3 μT for brain abscess and tumor, respectively. This relationship was preserved when tested with two different tumor models in rats (specimens were injected with F98 or 9 L glioma cell lines). The gluCEST signal has also successfully distinguished between encephalitis and ischemic lesions, where glutamate concentrations are found to be decreased in lacunar infarction regions but increased in encephalitis lesions [46].

A few studies have been published regarding diagnostic investigation using gluCEST to image the destruction of the blood–brain barrier by brain abscess in SD rat specimens [4]. This preliminary study showed that gluCEST may diagnose abscesses more reliably and at a higher resolution than MRS [4]. Another study explored the use of gluCEST imaging in vivo in rats and humans to diagnose encephalitis [46]. In model rats, glutamate concentrations were significantly associated with the inflammation caused by *S. aureus*-induced encephalitis lesions; furthermore, the gluCEST signal increased throughout the infection. This suggests that further studies could investigate how changes in glutamate concentrations could be used to monitor encephalitis development throughout the infection [46]. Another study used gluCEST imaging to examine the detection of early changes occurring in sepsis-associated encephalopathy (SAE), a severe systemic infection complication affecting the CNS [47]. Unlike the use of conventional MRI, combining gluCEST with diffusion kurtosis imaging (DKI) could detect early abnormalities in diseased rat cohorts [47]. Lastly, another group investigated the glutamate signal changes during SAE within the hippocampus in SD rats using gluCEST [48]. In this study, H1-MRS imaging was used to understand if CEST could provide high spatial resolution for quantitative brain mapping and lesion visualization in SAE. The results found that gluCEST imaging could provide sensitive data regarding changes in glutamate in the hippocampus of the rats with SAE with comparable quality to H1-MRS imaging [48].

**Figure 2:**
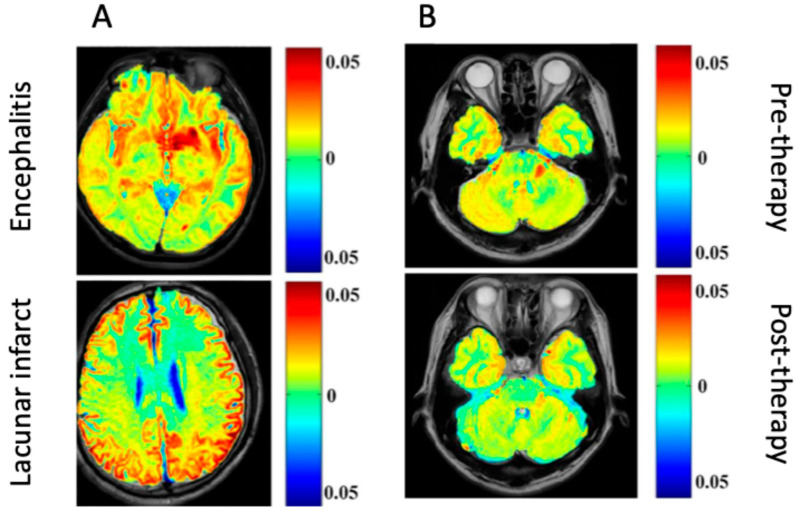
Glutamate chemical exchange saturation transfer (gluCEST) in patients. (**A**) Differential images comparing encephalitis and lacunar infarct. (**B**) Images showing encephalitis before and after immunoglobulin therapy [46] (Images obtained from an open-access article distributed under the terms of the Creative Commons Attribution License (CC BY)).

CEST MRI can also be used to visualize the infections of the CNS over time, essentially monitoring their status and providing information on prognosis and therapeutic efficacy. In rat models, bacCEST can monitor antibiotic therapy response in brain abscesses. This visualization method was also superior to structural MRI, as the bacCEST contrast showed a change much earlier than T2WI (after 4 days instead of 10 days) (shown in Figure 2 as an illustration) [45]. Another preclinical study investigated the effects of N-acetylcysteine (NAC) treatment on SAE in SD rats [47]. The gluCEST signal was significantly higher in the SAE group than in the NAC-treated group or the controls, and the CEST distribution also differed in the SAE group. These findings suggest that the effects of treatment on the cortex could be visualized through gluCEST imaging in this disease process.

Another emerging research field uses CEST for the theranostic treatment of CNS pathologies with virulent NPs. One research group developed a theranostic technology involving CEST for the treatment of HIV encephalitis [42]. In this study, the CEST signal of antiretroviral therapy (ARV) cytidine analogs were characterized in vivo in Male C57BL/6 mice using hydroxyl and amino protons found in their chemical structure. This application of CEST could allow for the biodistribution of HIV drugs to be tracked through sub-regions of the brain and linearly correlated with their concentration. This could be used to understand which areas of the CNS these therapies cannot reach and the mechanisms of ARV efficacy and toxicity [42].

**Table 2:** Preclinical studies performed with CEST.

Strain	Type of Infection	Animal Model	MRI Scanner Used	Type of CEST	Findings	Reference
CN	Monitoring treatment with tumor-homing bacteriolytic therapy	F444 Fisher rats (6 weeks old, female)	11.7 T Bruker Biospec system (Bruker Biosciences, Billerica, MA, USA)	bacCEST	- CEST contrast was at maximum at 2.6 ppm for bacterial suspensions in vitro; no contrast was obtained from spores - CEST contrast was significantly increased in *C. novyi*-NT infected tumors compared to tumors before bacterial germination and infection; CEST contrast was specific to this bacterium, not just inflammation - This type of CEST MRI can be achieved at 3 T field strength	[45]
HIV	Encephalitis—Developed HIV theranostics using CEST contrasts of ARVs—Traced drug biodistribution in brain sub-regions using CEST MRI	Male C57BL/6 mice (14–16 weeks old)	7.0 T scanner (Bruker PharmaScan 70/16, Billerica, MA, USA)	CEST based on the contrasts of ARV drugs	- The CEST effects of ARV cytidine analogs (using their hydroxyl proton and amine proton) can be characterized in vivo in different sub-regions of the brain with MRI - CEST effects linearly correlated to drug concentrations in the brain - Possibility for use in in vivo detection and quantification in humans using advanced data analysis algorithms like Lorentzian line-shape fitting	[42]
HS	Monitoring of oncolytic herpes simplex virus G47Δ through MR imaging reporter gene known as lysine-rich protein (LRP)	Fisher-344 rats	9.4 T Bruker BioSpin (Bruker Biosciences, Billerica, MA, USA)	Multiple CEST frequencies (−2100 to +2100 Hz)	- The hybrid G47Δ-LRP virus had the same therapeutic effectivity as G47Δ-empty virus - CEST contrast was observed in cancer cells and tumors after infection with G47Δ-LRP virus, not with G47Δ-empty viruses	[49]
SA	To differentiate abscesses from brain tumors	Rat–female	11.7 T Bruker Biospec system (Bruker Biosciences, Billerica, MA, USA)	bacCEST	- BacCEST signal of *S. aureus* was characterized as a strong and broad signal in the range of 1–4 ppm (max at 2.6 ppm) arising from all proton-exchangeable components of bacteria (e.g., carbohydrates, peptides, proteins, metabolites) - CEST signal in *S. aureus*-induced brain abscesses was significantly higher than the rest of the brain parenchyma - BacCEST contrast decreased significantly after 4 days of treatment, earlier than the comparative T2-weighted image (10 days) - BacCEST could effectively differentiate brain abscess from tumor using ΔCEST	[45]
	Brain abscess—Changes in glutamate levels in brain abscess	SD rats (female)	Agilent 7 T animal MRI scanner (Agilent Technology, Inc., Santa Clara, CA, USA)	gluCEST	- A significant increase in GLUCEST signal was observed in infective lesions - Increased glutamate concentration (in %) was observed in rats with brain abscesses - Higher GluCEST contrast was observed in GM relative to WM	[4]
	Encephalitis—Differentiation of encephalitis lesions from lacunar infarction	SD rats (adult)	Agilent 7 T MR scanner (Agilent Technology, Inc., Santa Clara, CA, USA)	gluCEST	- The number of amine protons with a gluCEST signal was proportional to glutamate concentration - Encephalitis areas showed hyper-intense gluCEST signal compared to normal tissue	[46]
SAE	Effect of NAC	SD rats	7.0 T MRI scanner (company not provided)	gluCEST	- GluCEST showed that Glu metabolism changes occurred early in SAE, where the related toxicity starts when Glu is over-released with insufficient synaptic clearance - The gluCEST signal was significantly higher in the SAE group than in the N-acetylcysteine-treated group or the controls - The CEST distribution throughout the brain also differed in the SAE group	[47]
	Sepsis-associated encephalopathy evaluating Glu signal changes with H1 MRS vs. gluCEST imaging	21 SD rats (8 weeks old)	7 T horizontal-bore PharmaScan 70/16 scanner (Bruker BioSpin GmbH, Ettlingen, Germany)	gluCEST	- Higher GluCEST and H-MRS values were observed in the sepsis-associated encephalopathy group than controls - gluCEST had a high sensitivity to changes in Glu signals in the hippocampus associated with SAE (higher compared to H1 MRS)	[48]

CN, *Clostridium novyi*-NT; HIV, human immunodeficiency virus; HS, herpes simplex; NAC, N-acetylcysteine; SA, *Staphylococcus aureus*; SAE, sepsis-associated encephalopathy.

Lastly, CEST has also been used to track infectious particles in the CNS when they are utilized as therapies for other pathologies; two different study groups have assessed oncolytic viral therapy for brain tumors. In the first one, a genetically modified, non-virulent bacterial strain (*C. novyi-NT*) demonstrated its ability to selectively infect experimental brain tumors and induce regression alone or with combination therapies [45]. BacCEST was used to non-invasively image the distribution, accumulation, and clearance of this injected bacterial therapy throughout the CNS, essentially inducing and monitoring a non-virulent and targeted infection as it occurred in F344 Fisher rat subjects. The results from this study showed that this type of endogenous CEST MRI enabled direct visualization of the tumor-homing bacteria and thus has immense potential for monitoring other *C. novyi-NT*-based cancer therapies and providing insight into diagnosing bacterial infections in the CNS. The other study involved engineering an MR imaging reporter gene into G47Δ, a herpes simplex-derived oncolytic virus [49]. The G47Δ-lysine rich protein (LRP) hybrid virus could be visualized through CEST MRI at each stage of acute viral infection and as it replicated within cancer cells to treat induced brain tumors. In CNS infection, this study exposes an intriguing possibility of using LRP as a reporter for the real-time detection of viral spread (such as in cases of clinical viral encephalitis or meningitis) [49].

### 7.2. Clinical Findings

In addition to CEST trials in animal models, there is also some preliminary research on the clinical usage of CEST in humans (Table 3). GluCEST MRI has detected encephalitis lesions in human patients with high sensitivity, even with a low magnetic strength scanner (3.0 T), which is commonly used in clinical practice [46]. Another clinical study compared the sensitivity of APT imaging versus Gd-T1w MRI while imaging various pediatric CNS infections [50]. The results found that the APTw contrast images agreed with the Gd-enhanced T1w MR images for detecting intracranial infectious lesions. Also, the study found that the APTw frequency values of infectious lesions were significantly lower than those of neoplastic lesions. The best cut-off for this delineation, with a sensitivity of 88.6% and specificity of 70.8%, was 2.30% [50].

The severity of lesions in encephalitis can also be tracked over time with gluCEST during intravenous immunoglobulin therapy. The gluCEST signal in encephalitis lesion areas was significantly decreased from pre- to post-treatment in human participants, which correlated with the signal intensity changes visualized on conventional MRI imaging [46]. However, the gluCEST signal change was specific to encephalitis lesions; lacunar infarction lesions treated with the same therapy could not be adequately visualized over time with gluCEST [46].

Lastly, APT (amide CEST) MRI can be used to differentiate types of infective mass and neoplastic lesions in the human CNS [51]. The number of infections isolated in the treatment of naïve human participants in this study were tuberculosis (*n* = 3), tubercular abscess (*n* = 1), *Aspergillus* granuloma (*n* = 1), and neurocysticercosis (*n* = 3). This study confirmed that APTw contrast is higher in neoplastic and infective lesions in humans, likely due to increased cellular protein and peptide content. There was no significant difference between these two divisions of intracranial mass lesions with some statistical normalizations; however, using Type-4 normalization (APTnegNAWM) and histogram parameters corresponding to ROI-2 allowed for the two groups (as well as inter-group variations in etiology) to be separated by APT-w imaging [51].

**Table 3:** Clinical studies performed with CEST.

Study	CNS Infection(s)	MRI Scanner Used	Type of CEST	Findings	References
Patients with encephalitis and LI, and healthy controls Differentiation of encephalitis lesions from lacunar infarction; monitoring of encephalitis response to immunoglobulin treatment	Clinical encephalitis (varied pathology)	Agilent 7 T MR scanner (Agilent Technology, Inc., Santa Clara, CA, USA)	gluCEST	- Encephalitis areas demonstrated a hyper-intense gluCEST signal, while lacunar infarction areas had decreased intensity - Patients with encephalitis lesions showed a decrease in gluCEST signal following IV immunoglobulin therapy - This change in the CEST signal was significantly different from a pre-treatment signal	[46]
Pediatric patients with ICMLs (ages 2 years to 190 months)—Comparison of APTw and Gd-T1w MR findings in various pediatric CNS infections	Abscess, viral encephalitis, meningitis	3 T MR scanner GE Revolution 256 scanner (GE Healthcare, Chicago, IL, USA)	APT (amide CEST)	- APTw contrast images agree with Gd-enhanced T1w MR images for the detection of intracranial infectious lesions - APTw values of infectious lesions were significantly lower than that of neoplastic lesions—best cut-off value = 2.30% - Only 6 of the encephalitis images showed up on either Gd-T1w or APTw images - Compared to Gd-T1w MRI, APTw was comparable, but the spatial resolution and signal-to-noise ratio were suboptimal compared to SE/FSE imaging (sequences used to scan in T2WI)	[50]
Treatment naïve patients Differentiation of infective tuberculosis, tubercular abscess, *Aspergillus* granuloma) from neoplastic mass lesions (low- and high-grade gliomas)	Infective mass lesions (varied pathologies)	3T whole body Inginia MRI system (Philips Healthcare, Best, The Netherlands)	APT (amide CEST)	- APT-w contrast was higher in ICMLs than in the rest of the brain, likely due to increased cellular protein/peptide content in lesion regions - There was no significant difference among ICMLs (glioma vs. infection) in some normalizations, but by using type-4 normalization (APTnegNAWM) and active lesion region (ROI-2) differentiation, the lesions could be delineated from one another	[51]

APT, amide proton transfer.

Generally, differences in the imaging between different events in the brain could provide a wrong diagnosis. For example, the difference between encephalitis and lacunar infarction could not provide a differential picture for an accurate diagnosis. However, a clinical study demonstrated an increase in the gluCEST signal in patients with encephalitis compared to a decreased gluCEST signal in patients with lacunar infarction [46]. To validate their finding, patients with encephalitis were treated with a cocktail of immunoglobulins, which caused a decrease in the encephalitis lesions (1.34% to 5%) (Figure 2).

## 8. Limitations

Despite these significant findings at the intersection of CEST MRI and neuroinfectious disease, many components of CEST imaging limit its potential for practical application. MRI scanners with high magnetic field strength (i.e., 7 T and above) are often used to investigate CEST imaging, particularly in animal studies. High magnetic fields allow for optimizing CEST physics properties with diaCEST by favoring slower exchange and increasing frequency separation [52]. Human brain imaging with 7 T scanners is not yet routinely available, with only two 7 T MR models approved for clinical use in the USA [53]. However, some publications in this review achieved significant CEST effects with 3 T scanners in patients. Researchers in this field will need to continue investigating how CEST effects can be obtained with more accessible scanners.

The standardization of CEST MRI experiments is also relatively unreliable; as discussed in the introduction, the detection of the exchanging group (many of which were discussed in this review) is susceptible to other factors such as pH or concentration. Therefore, comparing findings from different research groups is challenging without the universal standardization and control of these factors between and within research studies. The interpretation of the CEST signal is also often impacted by other tissue parameters such as direct water saturation (DS), nuclear overhauser enhancement effect (NOE, the change in intensity of a NMR resonance due to the irradiation of another resonance that is close in space), water longitudinal relaxation time (T1w), and the overlapping CEST effects of other molecules [46]. These challenges are currently being studied and require further analysis to determine the extent of their impact on CEST imaging data.

## 9. Future Research

The use of CEST for imaging neuroinfectious disease is a novel concept, and the breadth of research published at this time is relatively limited. This field would benefit from more studies investigating using CEST imaging with low magnetic strength scanners (e.g., 3 T machines) to understand how this technique could be applied to clinical medicine. In addition, more clinical studies must be performed to grasp better the quality of images that can be gathered from patients with neuroinfectious diseases. This work will inform future policy on using CEST MRI in acute or tertiary care settings and will also indicate which neuroinfectious diseases CEST is more suited towards imaging. Next, there was little to no data surrounding infections in the CNS that are not located in the brain’s parenchyma; it would be prudent for future data collection to focus on the possibility of CEST MRI to assess meningitis and subdural/epidural empyema.

## 10. Conclusions

This review assessed the status of the literature surrounding the use of CEST MRI in the field of neuroinfectious disease. The results show that most of the research in this area has focused on infections of the brain parenchyma (i.e., encephalitis) and that most studies investigate the use of CEST in animal models, with a minority exploring the application of CEST to human participants. The common neuroinfectious disease presentations relevant to clinical medicine were also briefly described, as well as the traditional and modern imaging techniques used to visualize them. We recommend that future works in this field explore the use of CEST for diagnosis and monitoring of human pathology; there is significant potential for CEST MRI to be used as a state-of-the-art imaging tool in modern clinical medicine.

## Figures and Tables

**Table 1:** Categories and characteristics of the types of CEST.

Category	Features	Mechanism	Subtypes
Paramagnetic	Typically exogenous. Contain metallic ions. More significant frequency shift than their diamagnetic counterparts (faster proton exchange)	Exchange drive by large chemical shift induced by a paramagnetic metal ion (typically lanthanide) in the contrast agent.	ParaCEST SupraCEST
Diamagnetic	Do not contain metallic ions. Can be endogenous, closer chemical shift to water (typically 0–7 ppm)	Exchange driven by the chemical shift of exchangeable protons on the contrast agent (either exogenous or endogenous)	LMW diaCEST-NH_4_Cl Macromolecular diaCEST–Poly-lysine, G-5 PAMAM dendrimer EndoCEST–Amide protons (APT), OH protons, amine protons, BacCEST, CryptoCEST

## References

[1] Wu B., Warnock G., Zaiss M., Lin C., Chen M., Zhou Z., Mu L., Nanz D., Tuura R., Delso G. (2016). An overview of CEST MRI for non-MR physicists. EJNMMI Phys..

[2] Erdem H., Inan A., Guven E., Hargreaves S., Larsen L., Shehata G., Pernicova E., Khan E., Bastakova L., Namani S. (2017). The burden and epidemiology of community-acquired central nervous system infections: A multinational study. Eur. J. Clin. Microbiol. Infect. Dis..

[3] Shah S., Turner M.L., Chen X., Ances B.M., Hammoud D.A., Tucker E.W. (2023). The promise of molecular imaging: Focus on central nervous system infections. J. Infect. Dis..

[4] Chen Y., Dai Z., Shen Z., Guan J., Zhuang Z., Mao Y., Mao R. (2018). Imaging of glutamate in brain abscess using GLUCEST at 7T. Radiol. Infect. Dis..

[5] Mullen M.P., Varghese G. (2016). Infections of the Nervous System. Mount Sinai Expert Guides.

[6] Ziai W.C., Lewin J.J. (2008). Update in the diagnosis and management of central nervous system infections. Neurol. Clin..

[7] Govic Y.L., Demey B., Cassereau J., Bahn Y.-S., Papon N. (2022). Pathogens infecting the central nervous system. PLoS Pathog..

[8] Riddell J., Shuman E.K. (2012). Epidemiology of central nervous system infection. Neuroimaging Clin. N. Am..

[9] Steigbigel N.H. (2001). Computed tomography of the head before a lumbar puncture in suspected meningitis—Is it helpful?. N. Engl. J. Med..

[10] Bolan G., Barza M. (1985). Acute bacterial meningitis in children and adults. A perspective. Med. Clin. N. Am..

[11] Kastrup O., Wanke I., Maschke M. (2005). Neuroimaging of infections. NeuroTherapeutics.

[12] Hunter J.V., Morriss M.C. (2003). Neuroimaging of central nervous system infections. Semin. Pediatr. Infect. Dis..

[13] Sharath Kumar G.G., Adiga C.P., Iyer P.P., Goolahally L.N. (2022). Role of imaging in CNS infections. Indian J. Pathol. Microbiol..

[14] Rangarajan K., Das C.J., Kumar A., Gupta A.K. (2014). MRI in central nervous system infections: A simplified patterned approach. World J. Radiol..

[15] Gasparetto E.L., Cabral R.F., da Cruz L.C.H., Domingues R.C. (2011). Diffusion imaging in brain infections. Neuroimaging Clin..

[16] Guglielmetti C., Veraart J., Roelant E., Mai Z., Daans J., Van Audekerke J., Naeyaert M., Vanhoutte G., Delgado y Palacios R., Praet J. (2016). Diffusion kurtosis imaging probes cortical alterations and white matter pathology following cuprizone induced demyelination and spontaneous remyelination. NeuroImage.

[17] Mahmud A. (2024). MR Spectroscopy|Radiology Reference Article|Radiopaedia.org. Radiopaedia. https://radiopaedia.org/articles/mr-spectroscopy-1.

[18] Wunder A., Klohs J., Dirnagl U. (2009). Non-invasive visualization of CNS inflammation with nuclear and optical imaging. Neuroscience.

[19] Burrell-Saward H., Ward T.H. (2016). Bioluminescence imaging to detect late-stage infection of African trypanosomiasis. J. Vis. Exp..

[20] van Zijl P.C.M., Yadav N.N. (2011). Chemical exchange saturation transfer (CEST): What is in a name and what isn’t?. Magn. Reson. Med..

[21] Ward K.M., Aletras A.H., Balaban R.S. (2000). A new class of contrast agents for MRI based on proton chemical exchange dependent saturation transfer (CEST). J. Magn. Reson..

[22] Soesbe T.C., Wu Y., Sherry A.D. (2013). Advantages of paramagnetic CEST complexes having slow-to-intermediate water exchange properties as responsive MRI agents. NMR Biomed..

[23] Zhou J., van Zijl P.C.M. (2006). Chemical exchange saturation transfer imaging and spectroscopy. Prog. Nucl. Magn. Reson. Spectrosc..

[24] Terreno E., Castelli D.D., Viale A., Aime S. (2010). Challenges for molecular magnetic resonance imaging. Chem. Rev..

[25] Baranyai Z., Tircsó G., Rösch F. (2020). The use of the macrocyclic chelator DOTA in radiochemical separations. Eur. J. Inorg. Chem..

[26] Aime S., Calabi L., Biondi L., De Miranda M., Ghelli S., Paleari L., Rebaudengo C., Terreno E. (2005). Iopamidol: Exploring the potential use of a well-established x-ray contrast agent for MRI. Magn. Reson. Med..

[27] Goffeney N., Bulte J.W.M., Duyn J., Bryant L.H., van Zijl P.C.M. (2001). Sensitive NMR detection of cationic-polymer-based gene delivery systems using saturation transfer via proton exchange. J. Am. Chem. Soc..

[28] Dagher A.P., Aletras A., Choyke P., Balaban R.S. (2000). Imaging of urea using chemical exchange-dependent saturation transfer at 1.5T. J. Magn. Reson. Imag..

[29] Kogan F., Hariharan H., Reddy R. (2013). Chemical exchange saturation transfer (CEST) imaging: Description of technique and potential clinical applications. Curr. Radiol. Rep..

[30] Hancu I., Dixon W.T., Woods M., Vinogradov E., Sherry A.D., Lenkinski R.E. (2010). CEST and PARACEST MR contrast agents. Acta Radiol..

[31] Sherry A.D., Woods M. (2008). Chemical exchange saturation transfer contrast agents for magnetic resonance imaging. Annu. Rev. Biomed. Eng..

[32] Jones C.K., Schlosser M.J., van Zijl P.C.M., Pomper M.G., Golay X., Zhou J. (2006). Amide proton transfer imaging of human brain tumors at 3T. Magn. Reson. Med..

[33] Ling W., Regatte R.R., Navon G., Jerschow A. (2008). Assessment of glycosaminoglycan concentration in vivo by chemical exchange-dependent saturation transfer (gagCEST). Proc. Natl. Acad. Sci. USA.

[34] Meng N., Huang Z., Jiang H., Dai B., Shen L., Liu X., Wu Y., Yu X., Fu F., Li Z. (2024). Glucose chemical exchange saturation transfer MRI for predicting the histological grade of rectal cancer: A comparative study with amide proton transfer-weighted and diffusion-weighted imaging. Insights Imaging.

[35] Chan K.W.Y., McMahon M.T., Kato Y., Liu G., Bulte J.W.M., Bhujwalla Z.M., Artemov D., van Zijl P.C.M. (2012). Natural D-glucose as a biodegradable MRI contrast agent for detecting cancer. Magn. Reson. Med..

[36] Nasrallah F.A., Pagès G., Kuchel P.W., Golay X., Chuang K.-H. (2013). Imaging brain deoxyglucose uptake and metabolism by glucocest MRI. J. Cereb. Blood Flow Metab..

[37] Roussel T., Frydman L., Le Bihan D., Ciobanu L. (2019). Brain sugar consumption during neuronal activation detected by CEST functional MRI at ultra-high magnetic fields. Sci. Rep..

[38] Cai K., Haris M., Singh A., Kogan F., Greenberg J.H., Hariharan H., Deter J.A., Reddy R. (2012). Magnetic resonance imaging of glutamate. Nat. Med..

[39] Mao Y., Zhuang Z., Chen Y., Zhang X., Shen Y., Lin G., Wu R. (2019). Imaging of glutamate in acute traumatic brain injury using chemical exchange saturation transfer. Quant. Imaging Med. Surg..

[40] Liu G., Bettegowda C., Qiao Y., Staedtke V., Chan K.W.Y., Bai R., Li Y., Riggins G.J., Kinzler K.W., Bulte J.W. (2013). Noninvasive imaging of infection after treatment with tumor-homing bacteria using Chemical Exchange Saturation Transfer (CEST) MRI. Magn. Reson. Med..

[41] Vanherp L., Poelmans J., Weerasekera A., Hillen A., Croitor-Sava A.R., Sorrell T.C., Lagrou K. (2021). Trehalose as quantitative biomarker for in vivo diagnosis and treatment follow-up in cryptococcomas. Transl. Res..

[42] Bade A.N., Gendelman H.E., McMillan J., Liu Y. (2021). Chemical exchange saturation transfer for detection of antiretroviral drugs in brain tissue. AIDS.

[43] Rahman M., Islam R., Akash S., Harun-Or-Rashid M., Ray T.K., Rahaman S., Islam M., Anika F., Hosain M.K., Aovi F.I. (2022). Recent advancements of nanoparticles application in cancer and neurodegenerative disorders: At a glance. Biomed. Pharmacoth..

[44] Dreifuss T., Betzer O., Shilo M., Popovtzer A., Motiei M., Popovtzer R. (2015). A challenge for theranostics: Is the optimal particle for therapy also optimal for diagnostics?. Nanoscale.

[45] Liu J., Bai R., Li Y., Staedtke V., Zhang S., van Zijl P.C.M., Liu G. (2018). MRI detection of bacterial brain abscesses and monitoring of antibiotic treatment using bacCEST. Magn. Reson. Med..

[46] Jia Y., Chen Y., Geng K., Cheng Y., Li Y., Qiu J., Huang H., Wang R., Zhang Y., Wu R. (2020). Glutamate chemical exchange saturation transfer (GluCEST) magnetic resonance imaging in pre-clinical and clinical applications for encephalitis. Front. Neurosci..

[47] Zhong Y., Guan J., Ma Y., Xu M., Cheng Y., Xu L., Lin Y., Zhang X., Wu R. (2023). Role of imaging modalities and N-acetylcysteine treatment in sepsis-associated encephalopathy. ACS Chem. Neurosci..

[48] Lee D.-W., Kwon J.-I., Heo H., Woo C.-W., Yu N.H., Kim K.W., Woo D.C. (2023). Cerebral glutamate alterations using chemical exchange saturation transfer imaging in a rat model of lipopolysaccharide-induced sepsis. Metabolites.

[49] Farrar C.T., Buhrman J.S., Liu G., Kleijn A., Lamfers M.L.M., McMahon M.T., Gilad A.A., Fulci G. (2015). Establishing the lysine-rich protein CEST reporter gene as a CEST MR imaging detector for oncolytic virotherapy. Radiology.

[50] Zhang H., Zhou J., Peng Y. (2021). Amide proton transfer–weighted MR imaging of pediatric central nervous system diseases. Magn. Reson. Imag. Clin..

[51] Debnath A., Gupta R.K., Singh A. (2020). Evaluating the role of amide proton transfer (APT)–weighted contrast, optimized for normalization and region of interest selection, in differentiation of neoplastic and infective mass lesions on 3T MRI. Mol. Imaging Biol..

[52] Vinogradov E., Sherry A.D., Lenkinski R.E. (2013). CEST: From basic principles to applications, challenges and opportunities. J. Magn. Reson..

[53] Okada T., Akasaka T., Thuy D.H., Isa T. (2022). Safety for human MR scanners at 7T. Magn. Res. Med. Sci..

